# Removal of a Nævus by a Platinum Wire, Heated by a Galvanic Current

**Published:** 1852-03

**Authors:** 


*Removal of a Nœvus by a Platinum Wire, heated by a Galvanic Current.*—Our readers probably remember the cases of fistula in ano and hæmorrhoids successfully treated by Mr. Marshall, at University College Hospital, with the platinum wire, made red hot by a galvanic battery. We perceive that Mr. Hilton has been trying this plan of cutting and searing at the same time upon a nævus of the flat kind, situated in front of the ear of a child two months old. The operation was performed with Cruikshank's battery and a very thin wire, which it was first intended to tie around half the tumor, which was about the size of a crown piece. But the wire ran so easily through it, that the whole was completely removed, and the parts are now fast cicatrizing. This is rather a quicker measure than the ligature, and just as secure, since hæmorrhage is so rare.—*Ibid.*

